# Pancraniosynostosis following endoscopic-assisted strip craniectomy for sagittal suture craniosynostosis in the setting of poor compliance with follow-up: a case report

**DOI:** 10.1186/s13256-015-0549-0

**Published:** 2015-03-24

**Authors:** Walavan Sivakumar, Isak Goodwin, Ross Blagg, Dana Johns, Jay Riva-Cambrin, Faizi Siddiqi, Barbu Gociman

**Affiliations:** 1 Department of Neurosurgery, Primary Children’s Hospital, University of Utah, 100 Mario Capecchi Drive, Salt Lake City, UT 84132 USA; 2 Department of Plastic Surgery, University of Utah Hospital, 30 North 1900 East, Suite 3B400, Salt Lake City, UT 84132 USA

**Keywords:** Helmet therapy, Strip craniectomy, Craniosynostosis, Pansynostosis, Plagiocephaly, Minimally invasive, Sagittal synostosis

## Abstract

**Introduction:**

There is limited craniofacial literature on the complications of helmet therapy and controversy regarding the effects of inadequate orthotic helmet therapy. The authors present a case of inadvertently prolonged orthotic helmet therapy after endoscopic strip craniectomy for isolated sagittal synostosis.

**Case presentation:**

A two-month-old Caucasian baby underwent uncomplicated endoscopic-assisted strip craniectomy to treat synostosis of the sagittal suture and was fitted for an orthotic helmet two weeks postoperatively. He presented to the craniofacial clinic eight weeks postoperatively with occipital flattening and increased posterior vault height, so the helmet was refitted. During the next 18 months, the helmet was used inconsistently without follow-up. Upon re-presentation, the patient had developed pansynostosis, requiring a subsequent open total cranial vault reconstruction for correction for this secondary deformity.

**Conclusions:**

Although it remains unclear whether postoperative development of pansynostosis is the result of prolonged helmeting or the consequence of progressive synostotic disease, this report highlights the importance of parent education and judicious scheduled follow-up for the avoidance of potential helmet therapy complications.

## Introduction

Orthotic helmet therapy is an accepted treatment of positional plagiocephaly, as well as of postoperative cranial molding after endoscopic strip craniectomy. While this is a relatively new technique, early analyses have shown that endoscopic strip craniectomy followed by postoperative helmet molding is an effective, safe, and durable treatment modality [1,2]. Despite the possibility of air emboli, cerebral parenchymal injuries, significant cerebrospinal fluid leaks, seizures, and the need for conversion to an open approach, adverse effects have been extremely rare [2]. Complications of helmet therapy have been described, including the development of pressure sores, local ethanol erythema (related to buildup of cleaning fluids at the helmet-skin interface), skin infection, subcutaneous abscess, unsatisfying fit affecting adherence to therapy, and failed correction of head deformity [3]. Our report documents postoperative development of pansynostosis in a patient who initially presented with an uncomplicated single-suture sagittal synostosis treated with endoscopic-assisted strip craniectomy and postoperative molding helmet therapy. We discuss the potential contributions of poor adherence to helmeting and the natural progression of synostotic disease in the development of postoperative pansynostosis.

## Case presentation

**Figure 1:**
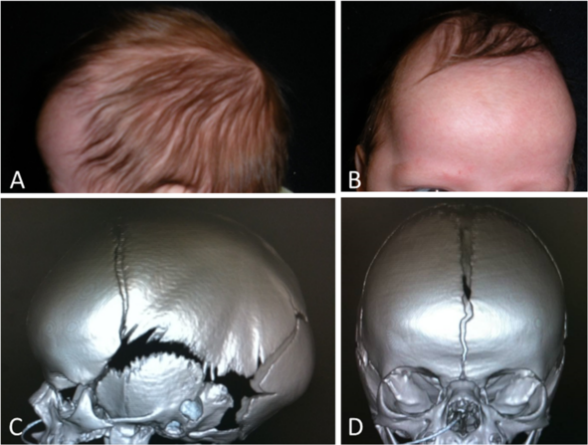
**Scaphocephaly from sagittal craniosynostosis.** Phenotypic photographs **(A, B)** and computed tomography imaging **(C, D)** showing preoperative scaphocephaly from sagittal craniosynostosis.

A three-week-old Caucasian male born at term was found to have an abnormally shaped head and subsequently was referred for craniofacial surgical evaluation. The 21-year-old mother’s pregnancy course was uncomplicated; she had routine prenatal care and an uncomplicated vaginal delivery. The physical examination demonstrated bifrontal bossing, a raised and thickened sagittal suture, and an elongated anteroposterior diameter skull, consistent with scaphocephaly secondary to sagittal synostosis (Figure 1A,B). Computed tomography (CT) imaging of the head confirmed synostosis of the sagittal suture, with the remaining sutures open (Figure 1C,D). The parents were counseled regarding treatment options. Both open and endoscopic-assisted surgical techniques were explained. In the case of the endoscopic procedure, the need for up to 12 months of postoperative helmet therapy was emphasized. The family opted to proceed with the endoscopic approach.

At two months of age, our patient underwent an uncomplicated endoscopic-assisted strip craniectomy with excision of an 11 × 5-cm strip of bone containing the fused suture, in accordance with our described technique [2]. He was fitted for an orthotic helmet two weeks postoperatively. The family failed to follow up with the orthotist, but presented to the craniofacial clinic eight weeks postoperatively. Our patient was noted to have occipital flattening and increased height of the posterior vault. At the time, the flattening was interpreted to be secondary to excess occipital helmet pressure, which could have been avoided by adherence to the routine monthly orthotic adjustments. Our patient was sent to the orthotist for refitting.

**Figure 2:**
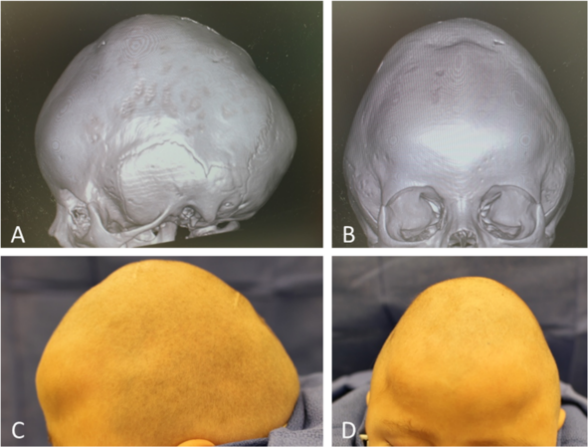
**Pansynostosis and oxycephaly.** Computed tomography images **(A, B)** and preoperative photos **(C, D)** for secondary corrective surgery showing pansynostosis and oxycephaly after inconsistent helmet therapy after endoscope-assisted strip craniectomy.

**Figure 3:**
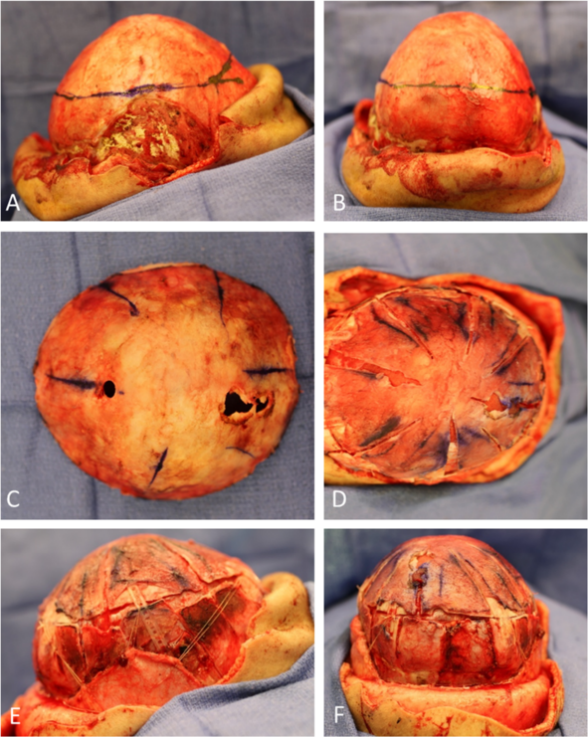
**Intraoperative photos of procedure to correct pansynostosis with oxycephaly after prolonged helmet therapy.** Preoperative markings of left lateral and anteroposterior views **(A, B)**; osteotomized skull vertex and barrel stave osteotomies **(C, D)**; completed cranial reconstruction with temporalis muscle flaps, left lateral and anteroposterior views **(E, F)**.

Our patient was lost to follow-up for the next six months. During this time the same helmet was inconsistently used, without follow-up with the craniofacial surgery team or the orthotist. According to our patient’s parent, a complicated social situation led to long periods in which the helmet use was discontinued altogether. Our patient next presented 18 months postoperatively. His head circumference was noted to be less than the first percentile for age, and his head shape was oxycephalic. The fontanelle was small, but patent and flat. Additionally, our patient was walking unsteadily and was slightly delayed regarding his language skills. A CT scan of his head showed pansynostosis (Figure 2). The brain parenchyma, ventricles, and cisterns were normal in appearance. Our patient underwent open cranial vault reconstruction (Figure 3A-F).

**Figure 4:**
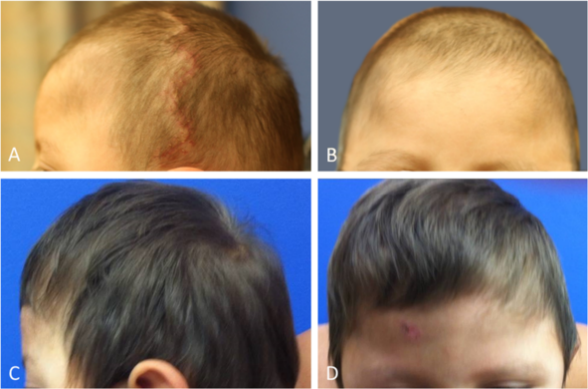
**Postoperative photos following open cranial vault reconstruction to repair pancraniosynostosis.** Left lateral and anteroposterior images at one month **(A, B)** and six months **(C, D)** after the secondary cranial reconstruction.

Postoperatively, our patient progressed well, having a good secondary result at the one-month follow-up visit (Figure 4A,B). At the six-month postoperative follow-up for the open cranial vault reconstruction, his head circumference had increased to the fifth percentile for age, and his head shape remained significantly improved (Figure 4C,D). In addition, no clinical signs or symptoms of increased intracranial pressure were noted.

## Discussion

Pansynostosis has been defined as the fusion of three or more cranial sutures [4]. Single-photon emission computed tomography (SPECT) studies have shown that craniosynostosis may be associated with decreased cerebral blood flow from the brain constriction, precipitated by premature suture fusion, and can lead to abnormal brain development. Correction of this synostosis allows for the normalization of cerebral blood flow and should be performed within the first eight months of life [5].

Infrequent cases have been described in which pansynostosis developed after cranial vault surgery or endoscopic-assisted strip craniectomy for craniosynostosis [6]. The incidence of additional suture synostosis developing postoperatively has been estimated to be 1 to 1.6% [7]. Postoperative pansynostosis occurs less frequently, with an incidence of 0.1% [6,8,9].

Despite the best efforts of the surgeon and craniofacial team, however, patients can be lost to follow-up, as in the case presented here. Our standard follow-up for all patients undergoing strip craniectomy and helmet remodeling therapy consists of evaluation immediately after surgery, and at three, six, nine, and twelve months after surgery. The follow-up is then continued on a yearly basis for up to five years. In addition to lack of adherence to the follow-up schedule, this patient used the postoperatively prescribed orthotic helmet sporadically. It is possible that the compressive force, albeit intermittent in this case, generated by an unadjusted helmet could have promoted the premature fusion of the remaining sutures. Alternatively, our patient’s subsequent suture fusion may have been the result of the natural progression of pansynostosis, independent of helmeting [10].

## Conclusions

This case illustrates the postoperative development of pansynostosis after endoscopic strip craniectomy. It is unclear whether this phenomenon is the result of inconsistent helmeting in the postoperative period or the effect of progressive nonsyndromic craniosynostotic disease. It is possible that our patient exhibited a familial suture synostosis or an atypical form of Crouzon syndrome [11]. The answer to this question will require further research into postoperative suture physiology in nonsyndromic craniosynostosis patients and the pathophysiology of pansynostosis. In addition, this report highlights the pitfalls of performing cranial vault reconstruction on patients with complex social situations that may hinder patient care. Close postoperative follow-up is essential to monitor for signs and symptoms consistent with progressive synostosis and/or raised intracranial pressure.

## Consent

Written informed consent was obtained from the patient’s legal guardian for publication of this case report and any accompanying images. A copy of the written consent is available for review by the Editor-in-Chief of this journal.

## Abbreviations

CT: computed tomography
SPECT: single-photon emission computed tomography
